# Methylglyoxal: A Relevant Marker of Disease Activity in Patients with Rheumatoid Arthritis

**DOI:** 10.1155/2018/8735926

**Published:** 2018-02-11

**Authors:** Ines Knani, Hassan Bouzidi, Saoussen Zrour, Naceur Bergaoui, Mohamed Hammami, Mohsen Kerkeni

**Affiliations:** ^1^Laboratory of Biochemistry, LR12ES05, Faculty of Medicine, University of Monastir, Monastir, Tunisia; ^2^Laboratory of Biochemistry, University Hospital Tahar Sfar, Mahdia, Tunisia; ^3^Department of Rheumatology, University Hospital of Fattouma Bourguiba, Monastir, Tunisia

## Abstract

**Background:**

The contribution of methylglyoxal (MGO) and soluble receptor for advanced glycation end products (sRAGE) in the presence of rheumatoid arthritis (RA) is still unknown. We investigated whether serum MGO and sRAGE were related to the presence of disease activity in RA.

**Methods:**

80 patients with RA and 30 control subjects were included in a cross-sectional study. The severity of RA was assessed using the disease activity score for 28 joints (DAS28). Serum MGO and sRAGE were measured by ELISA.

**Results:**

Serum MGO levels were significantly higher in patients with RA versus control subjects (*P* < 0.001) and were increased in RA patients with higher disease activity versus RA patients with moderate disease activity (*P* = 0.019). Serum sRAGE concentrations were significantly decreased in RA patients with higher disease activity versus RA patients with moderate disease activity and versus control subjects (*P* = 0.004; *P* = 0.002, resp.). A multiple logistic regression analysis demonstrated that MGO was independently associated with the presence of activity disease in RA (OR = 1.17, 95% CI: 1.02–1.31, *P* = 0.01).

**Conclusion:**

Serum MGO and sRAGE levels are inversely related to the activity of RA, and MGO is independently associated with a higher disease activity of RA.

## 1. Introduction

Rheumatoid arthritis (RA) is a serious health problem and is the most common form of chronic inflammatory rheumatism with an estimated prevalence of between 0.3 and 1% of the general adult population [1]. The development of this chronic disease is characterized by synovial joint inflammation, overgrowth of synoviocytes, progressive erosions, and cartilage destruction [2, 3]. RA may lead to joint destruction, causing impairment of the quality of life, disability, and reduced life expectancy, on average estimated at 10 years [4, 5]. The etiology of the disease remains unknown, and knowledge of the pathogenesis of RA has increased significantly in recent years [6]. More patients have moderate to high disease activity, and some develop aggressive systemic complications and joint damage. We showed previously that advanced glycation end products (AGEs), formed by a nonenzymatic glycation process, have been involved in many diseases such as diabetic microvascular and macrovascular diseases, atherosclerosis, and metabolic syndrome [7–10]. Recently, in RA disease, we demonstrated that *Nε*-carboxymethyllysine (CML) and pentosidine, as AGEs, were increased during RA. Again, we showed that CML was independently associated with the presence and the severity of RA [11].

Methylglyoxal (MGO), one of the reactive carbonyl species, formed endogenously as a by-product of the glycolytic pathway by degradation of triosephosphates or nonenzymatically by sugar fragmentation reactions, is the most potent precursor of AGE formation, in particular pentosidine formation [12, 13]. MGO is able to induce cellular damage, cross-linking of proteins, and glycation, playing an important role in the pathogenesis of many diseases [14]. With respect to AGE measurement, it is more interesting to evaluate the relationships between the precursors of AGEs and the development of RA disease. In addition, the interaction of AGEs with their RAGE plays a major role in inducing the inflammatory process [15, 16].

To shed light, no data exists regarding the contribution of MGO and sRAGE in the presence of RA; for that, we measured serum MGO and sRAGE levels in RA patients, and we examined whether these biomarkers are related to the severity of RA.

## 2. Patients and Methods

### 2.1. Study Population

The study protocol was approved by the regional ethical research committee. Written informed consent was obtained from all patients before the enrolment. This study includes 110 participants: RA group: eighty patients with RA diagnosed according to the American Rheumatism Association criteria [17] and without cardiovascular disease, nephropathy, or retinopathy were recruited in the rheumatology department in the University Hospital of Fattouma Bourguiba, Monastir (Tunisia). Control group: thirty healthy volunteers were included; they were matched with the patients for age and sex.

### 2.2. Biochemical Investigations

Blood samples of all participants (patients and controls) were collected in the morning, after an overnight fast (12 h), and then samples were stored at −80°C until analysis. Serum glucose, serum creatinine, uric acid, total cholesterol, triglyceride, HDL, and LDL were measured using the enzymatic method (CX9 autochemical analysis instrument, Beckman, USA). Glycated hemoglobin A1C (HbA1C) was measured using a G7 HPLC analyser (Tosoh Europe NV). High-sensitivity C-reactive proteins (hs-CRPs) were quantified according to the instructions of manufacturers using the particle-enhanced immunonephelometric method (BN II, Dade Behring). Anticyclic citrullinated peptides (anti-CCPs) were measured by a second-generation ELISA (Euroimmun®, Germany). The IgM rheumatoid factor (IgM-RF) was measured by the Waaler–Rose and latex agglutination tests (Fumouze®, France). MGO and sRAGE were measured in serum using ELISA kits provided by the YH Biosearch laboratory (Shanghai, China). MGO (or sRAGE) present in the sample was probed with an anti-MGO (or anti-sRAGE) antibody, followed by a horseradish peroxidase-conjugated secondary antibody. The MGO or sRAGE content in the sample was determined by comparison with a standard curve prepared from MGO standards ranging from 0 to 320 ng/mL or from sRAGE standards ranging from 0 to 12 ng/mL. The absorbance of the final color product was read at 450 nm by a Bio-Rad microplate reader (Model 680).

### 2.3. Disease Activity Assessment

Disease activity was used to assess the disease activity score for 28 joints (DAS28 score) according to EULAR's recommendations [18]. Ranges of the DAS28 score correspond with disease activity. A DAS28 score of <2.6 indicates remission. A DAS28 score of 2.6 to 3.2 indicates low disease activity. A DAS28 score of 3.2 to 5.1 indicates moderate disease activity. A DAS28 score of above 5.1 is considered high disease activity. Disability was assessed using the Health Assessment Questionnaire (HAQ), a score by questionnaire that examines the disabilities that RA patients encounter in daily living and activities [19]. The final HAQ index ranges from 0 to 3; HAQ scores < 0.3 are considered normal [20].

### 2.4. Statistical Analysis

All analyses were performed using SPSS version 17.0 (SPSS Inc., Chicago, Illinois, USA). Continuous variables were expressed as mean ± SD or median (interquartile range), while percentages were used to express categorical variables. An ANOVA test was used to compare the continuous variables between subgroups. An unpaired Student *t*-test was used for normally distributed variables, and the Mann–Whitney *U* test was used for skewed variables. To determine the independent parameters correlated with the presence of RA, the parameters that correlated significantly in the univariate analysis were tested using multiple backward stepwise regression analysis. A logistic regression analysis was performed to assess the association between MGO and sRAGE and the presence of RA. A receiver operating characteristic (ROC) curve analysis was performed to identify the optimal cut-off points of serum MGO or sRAGE levels for predicting RA. The area under the curve value was calculated to determine the accuracy of the test. *P* < 0.05 was considered as statistically significant.

## 3. Results

### 3.1. Clinical Characteristics of the Studied Population

The clinical characteristics and laboratory data of the control subjects and RA patients are shown in Table 1. Disease activity was measured by the DAS28 score (5.5 ± 1.3) at the time of investigation. 41% of all RA patients had moderate disease activity, and 59% had high disease activity. The mean ESR rate was 39.6 ± 27.2 mm (first hour). Anti-CCP and IgM-RF were present in 62.5% and 68.7% of the cases, respectively.

### 3.2. Biological Parameters of Patients with RA and Control Subjects

The biological parameters of patients and control subjects are shown in Table 2. Significant differences in body mass index and hs-CRP were seen between the groups. Triglyceride levels were significantly higher in RA patients compared to control subjects (*P* < 0.001), whereas their serum HDL-cholesterol levels were significantly lower compared to control subjects (*P* < 0.001). Patients showed an increased level of MGO (*P* < 0.001) and a decreased level of sRAGE (*P* = 0.020) compared to control subjects.

### 3.3. Relationship between Serum MGO and sRAGE Concentrations and the Presence of RA

**Figure 1:**
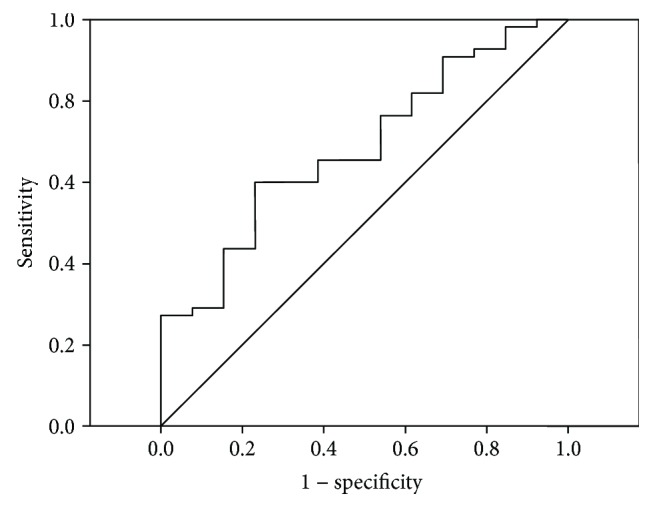
Receiver-operating characteristic (ROC) curve analysis for predictive values of serum concentrations of MGO and activity disease in RA. The area under the ROC curve is 0.69; 95% CI: 0.54–0.84, *P* = 0.019. The optimal cut-off value of MGO was 104 ng/mL, with 65% sensitivity and 62% specificity.

Multiple logistic regression analysis was performed using the presence of RA as a dependent variable. The analysis involved age, sex, BMI, glucose, triglyceride, HDL cholesterol, hs-CRP, MGO, and sRAGE. As a result, we find that serum MGO (OR = 1.17, 95% CI: 1.02–1.31, *P* = 0.01) was an independent predictor of the presence of RA. The ability of serum MGO concentrations to distinguish patients with RA from those without RA was assessed using ROC curve analysis. The ROC curves for the presence of RA diagnosis had an area under the curve (AUC) of 0.69 (95% CI: 0.54–0.84, *P* = 0.019), and the optimal cut-off value of MGO to predict the presence of activity disease in RA was 104 ng/mL, with 65% sensitivity and 62% specificity (Figure 1).

### 3.4. Relationship between Serum MGO and sRAGE Concentrations and Severity of RA

**Figure 2:**
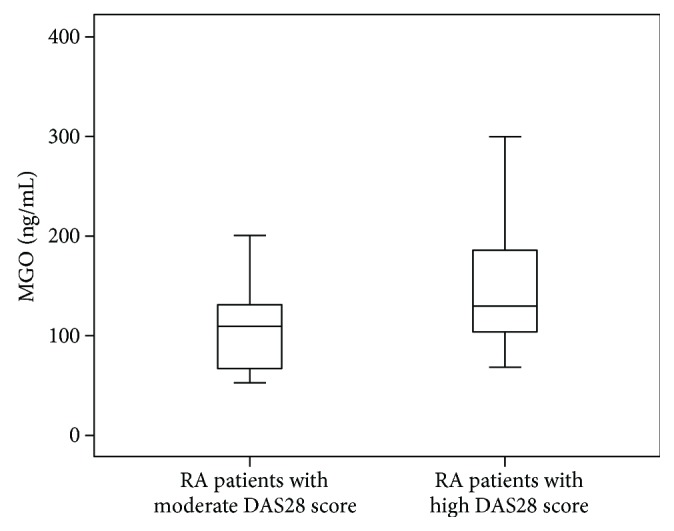
Serum concentrations of MGO in patients with moderate and high DAS28 scores (*P* = 0.019). The error bars represent minimum and maximum values; the boxes indicate the interquartile range and the median values.

**Figure 3:**
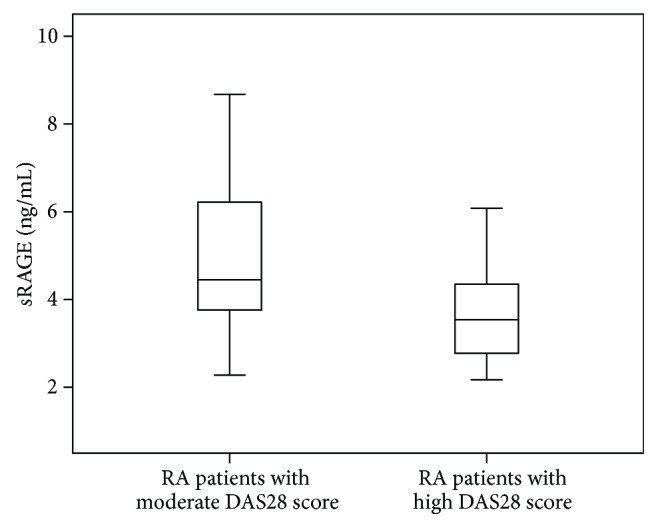
Serum concentrations of sRAGE in patients with moderate and high DAS28 scores (*P* = 0.004). The error bars represent minimum and maximum values; the boxes indicate the interquartile range and the median values.

All patients were subclassified into 2 subgroups according to DAS28. Serum concentrations of MGO were increased in patients with a high DAS28 score compared to patients with a moderate DAS28 score [129 (103–188), 109 (66–142); *P* = 0.019, resp.], as illustrated in Figure 2. Serum concentrations of sRAGE were decreased in patients with a high DAS28 score compared to patients with a moderate DAS28 score [3.5 (2.6–4.3), 4.4 (3.7–6.2); *P* = 0.004, resp.], as illustrated in Figure 3.

## 4. Discussion

AGE levels are increased in diabetes [7, 8], atherosclerosis [21, 22], nonalcoholic steatohepatitis [23], and neuroinflammatory diseases such as Alzheimer's disease [24] and Parkinson's disease [25]. Also, it has been demonstrated that increased AGE levels are correlated with the development of future microvascular and macrovascular events in diabetics and nondiabetics [8–10]. Recently, we have shown that CML and pentosidine were increased during RA and we demonstrated that CML was independently associated with the presence and the severity of RA [11].

MGO, one of the AGE precursors, is considered the most important source of AGE since it rapidly reacts with proteins to form AGE [26, 27]. Numerous studies revealed that the plasma MGO level is significantly increased in diabetic patients and is involved in diabetes-related vascular disorders such as hypertension, nephropathy, and retinopathy [28–31]. Recently, it indicated that MGO-derived hydroimidazolone-1 evokes inflammatory reactions in human umbilical vein endothelial cells via RAGE [32]. In our study, we showed an increased MGO level in RA patients and these levels were markedly increased in patients with high activity disease. MGO may induce and aggravate the severity in RA by a process that includes NF-*κ*B activation and the JNK system. A previous *in vivo* study showed that MGO was found to induce NF-*κ*B activation of murine peritoneal macrophages in sarcoma 180-bearing mice [28]. In an *in vitro* study, MGO activated the NF-*κ*B nuclear translocation in HIG-82 synovial cells using an immunofluorescent method [33]. MGO could be a stimulant for vascular inflammatory responses in cultured human vascular endothelial cells by MGO, which was mediated via the activation of JNK and p38 MAP kinase[34].

Our study revealed again not only decreased serum sRAGE levels in RA patients but also markedly decreased levels in patients with high DAS28 activity. RAGE, a multiligand cell surface protein, is a member of the immunoglobulin (Ig) superfamily. RAGE participates in inflammatory and immune responses, inducing leukocyte recruitment. The interaction between AGEs and the RAGE receptor, the most characterized receptor of AGEs, activates intracellular cells and secretes cytokines such as TNF-*α* which plays an important role in inflammatory response[35,36]. RAGE exists in soluble form; sRAGE is a truncated form of RAGE that serves as a decoy and acts as an anti-inflammatory molecule and may act as a receptor by binding ligands (such as AGEs) and preventing them from interacting with RAGE [37–39]. Previous studies have shown that RA patients have lower levels of sRAGE compared to healthy subjects [40–42]. Recently, overexpression of sRAGE in mesenchymal stem cells optimizes their immunoregulatory properties and may be useful as a novel cellular therapy for RA [43].

## 5. Conclusion

Patients with RA showed increased levels of MGO, and these levels were markedly elevated in line with the activity of RA. On the contrary, patients with high disease activity showed a significant decrease in sRAGE levels. Therapeutic ways of AGE development and precursor synthesis should be taken into account, and further experimental and *in vitro* studies merit advanced research for target molecule drug development.

## Figures and Tables

**Table 1:** Characteristics of patients with rheumatoid arthritis (RA) and control subjects.

	RA patients (*n* = 80)	Control subjects (*n* = 30)	*P*
Age (yrs)	52.9 ± 10.6	50.1 ± 8	NS
Female (%)	89	87	NS
Diabetes (%)	12	—	—
Hypertension (%)	20	—	—
Dyslipidemia (%)	64	—	
Erythrocyte sedimentation rate (ESR) (mm/H)	39.6 ± 27.2	9.8 ± 3.2	*P* < 0.001
Disease duration (yrs)	11.7 ± 8.2		
Swollen joint count (SJC)	2.4 ± 4.3		
Tender joint count (TJC)	16 ± 10		
HAQ score	1.5 ± 0.9		
Positive anticyclic citrullinated peptides (anti-CCPs, %)	62.5		
Positive IgM rheumatoid factor (%)	68.7		
DAS28	5.5 ± 1.3		
Remission (%)	0		
Low (%)	0		
Moderate (%)	41		
Active (%)	59		
Medication			
Methotrexate (%)	85		
Arava (%)	8.7		
Salazopyrin (%)	12.5		

Data represent means ± SD or percentages. NS: not significant.

**Table 2:** Biological parameters of patients with rheumatoid arthritis (RA) and control subjects.

	RA patients (*n* = 80)	Control subjects (*n* = 30)	*P*
BMI (kg/m^2^)	29.9 ± 7.6	24.8 ± 4.3	<0.001
hs-CRP (mg/L)	9 (5–15)	0 (0–5)	<0.001
Glucose (mmol/L)	4.5 ± 1.7	4.3 ± 0.2	NS
Triglyceride (mmol/L)	1.6 (1.1–2.1)	1.1 (0.8–1.4)	<0.001
Total cholesterol (mmol/L)	4.3 (3.7–4.8)	4.4 (4.1–5.3)	NS
HDL cholesterol (mmol/L)	1.3 (1.1–1.5)	0.9 (0.8–1.2)	0.001
LDL cholesterol (mmol/L)	2.5 (2.1–3)	2.7 (2.4–3)	NS
Creatinine (*μ*mol/L)	64.6 ± 23.6	64.5 ± 19.3	NS
MGO (ng/mL)	137.6 ± 76.6	80.4 ± 25.2	<0.001
sRAGE (ng/mL)	4.1 ± 1.4	5.4 ± 1.7	0.020

Data represent means ± SD or median (quartile range). NS: not significant.

## Abbreviations

AGEs: Advanced glycation end products
Anti-CCP: Anticyclic citrullinated peptides
BMI: Body mass index
CML: *Nε*-carboxymethyllysine
DAS28: Disease activity score for 28 joints
ESR: Erythrocyte sedimentation rate
HAQ: Health Assessment Questionnaire
HbA1C: Glycated hemoglobin A1C
HDL: High-density lipoprotein
hs-CRP: High-sensitivity C-reactive protein
IgM-RF: Immunoglobulin M rheumatoid factor
JNK: Jun *N*-terminal kinase
LDL: Low-density lipoprotein
MAP: Mitogen-activated protein kinase
MGO: Methylglyoxal
NF-*κ*B: Nuclear factor-kappa B
RA: Rheumatoid arthritis
RAGE: Receptor for advanced glycation end products
ROC: Receiver operating characteristic
sRAGE: Soluble receptor for advanced glycation end products
TNF-*α*: Tumor necrosis factor alpha.

## References

[1] Guillemin F., Saraux A., Guggenbuhl P. (2005). Prevalence of rheumatoid arthritis in France: 2001. *Annals of the Rheumatic Diseases*.

[2] FitzGerald O., Soden M., Yanni G., Robinson R., Bresnihan B. (1991). Morphometric analysis of blood vessels in synovial membranes obtained from clinically affected and unaffected knee joints of patients with rheumatoid arthritis. *Annals of the Rheumatic Diseases*.

[3] Iguchi T., Ziff M. (1986). Electron microscopic study of rheumatoid synovial vasculature. Intimate relationship between tall endothelium and lymphoid aggregation. *The Journal of Clinical Investigation*.

[4] Minaur N. J., Jacoby R. K., Cosh J. A., Taylor G., Rasker J. J. (2004). Outcome after 40 years with rheumatoid arthritis: a prospective study of function, disease activity, and mortality. *The Journal of Rheumatology Supplement*.

[5] Dadoun S., Zeboulon-Ktorza N., Combescure C. (2013). Mortality in rheumatoid arthritis over the last fifty years: systematic review and meta-analysis. *Joint, Bone, Spine*.

[6] Scott L., Wolfe F., Huizinga T. W. (2010). Rheumatoid arthritis. *The Lancet*.

[7] Kerkeni M., Saïdi A., Bouzidi H., Ben Yahya S., Hammami M. (2012). Elevated serum levels of AGEs, sRAGE, and pentosidine in Tunisian patients with severity of diabetic retinopathy. *Microvascular Research*.

[8] Kerkeni M., Saïdi A., Bouzidi H., Letaief A., Ben Yahia S., Hammami M. (2013). Pentosidine as a biomarker for microvascular complications in type 2 diabetic patients. *Diabetes and Vascular Disease Research*.

[9] Kerkeni M., Weiss I. S., Jaisson S. (2014). Increased serum concentrations of pentosidine are related to presence and severity of coronary artery disease. *Thrombosis Research*.

[10] Haddad M., Knani I., Bouzidi H., Berriche O., Hammami M., Kerkeni M. (2016). Plasma levels of pentosidine, carboxymethyl lysine, soluble receptor for advanced glycation end products, and metabolic syndrome: the metformin effect. *Disease Markers*.

[11] Knani I., Bouzidi H., Zrour S., Bergaoui N., Hammami M., Kerkeni M. (2017). Increased serum concentrations of N*ξ*-carboxymethyllysine are related to presence and severity of rheumatoid arthritis. *Annals of Clinical Biochemistry*.

[12] Thornalley J. (1996). Pharmacology of methylglyoxal: formation, modification of proteins and nucleic acids, and enzymatic detoxification—a role in pathogenesis and antiproliferative chemotherapy. *General Pharmacology*.

[13] Nasiri R., Field M. J., Zahedi M., Moosavi-Movahedi A. A. (2012). Comparative DFT study to determine if *α*-oxoaldehydes are precursors for pentosidine formation. *Journal of Physical Chemistry A*.

[14] Thornalley J. (2005). Dicarbonyl intermediates in the Maillard reaction. *Annals of the New York Academy of Sciences*.

[15] Schmidt A. M., Yan S. D., Yan S. F., Stern D. M. (2001). The multiligand receptor RAGE as a progression factor amplifying immune and inflammatory responses. *The Journal of Clinical Investigation*.

[16] Basta G., Lazzerini G., Massaro M. (2002). Advanced glycation end products activate endothelium through signal-transduction receptor RAGE: a mechanism for amplification of inflammatory responses. *Circulation*.

[17] Arnett F. C., Edworthy S. M., Bloch D. A. (1988). The American Rheumatism Association 1987 revised criteria for the classification of rheumatoid arthritis. *Arthritis and Rheumatism*.

[18] Fransen J., van Riel P. L. (2005). The disease activity score and the EULAR response criteria. *Clinical and Experimental Rheumatology*.

[19] Goh Y., Cooper M. E. (2008). Clinical review: the role of advanced glycation end products in progression and complications of diabetes. *The Journal of Clinical Endocrinology and Metabolism*.

[20] Kumar A., Malaviya A. N., Pandhi A., Singh R. (2002). Validation of an Indian version of the Health Assessment Questionnaire in patients with rheumatoid arthritis. *Rheumatology*.

[21] van Eupen M. G., Schram M. T., Colhoun H. M. (2013). The methylglyoxal-derived AGE tetrahydropyrimidine is increased in plasma of individuals with type 1 diabetes mellitus and in atherosclerotic lesions and is associated with sVCAM-1. *Diabetologia*.

[22] Hanssen N. M., Wouters K., Huijberts M. S. (2014). Higher levels of advanced glycation endproducts in human carotid atherosclerotic plaques are associated with a rupture-prone phenotype. *European Heart Journal*.

[23] Gaens K. H., Niessen P. M., Rensen S. S. (2012). Endogenous formation of N*ε*-(carboxymethyl)lysine is increased in fatty livers and induces inflammatory markers in an in vitro model of hepatic steatosis. *Journal of Hepatology*.

[24] Ahmed N., Ahmed U., Thornalley P. J., Hager K., Fleischer G., Munch G. (2005). Protein glycation, oxidation and nitration adduct residues and free adducts of cerebrospinal fluid in Alzheimer’s disease and link to cognitive impairment. *Journal of Neurochemistry*.

[25] Dalfo E., Portero-Otin M., Ayala V., Martinez A., Pamplona R., Ferrer I. (2005). Evidence of oxidative stress in the neocortex in incidental Lewy body disease. *Journal of Neuropathology and Experimental Neurology*.

[26] Thornalley P. J. (2008). Protein and nucleotide damage by glyoxal and methylglyoxal in physiological systems – role in ageing and disease. *Drug Metabolism and Drug Interactions*.

[27] Shiraki M., Kuroda T., Tanaka S., Saito M., Fukunaga M., Nakamura T. (2008). Nonenzymatic collagen cross-links induced by glycoxidation (pentosidine) predicts vertebral fractures. *Journal of Bone and Mineral Metabolism*.

[28] Wu L. (2006). Is methylglyoxal a causative factor for hypertension development?. *Canadian Journal of Physiology and Pharmacology*.

[29] Lapolla A., Flamini R., Dalla Vedova A. (2003). Glyoxal and methylglyoxal levels in diabetic patients: quantitative determination by a new GC/MS method. *Clinical Chemistry and Laboratory Medicine*.

[30] Wang X., Desai K., Chang T., Wu L. (2005). Vascular methylglyoxal metabolism and the development of hypertension. *Journal of Hypertension*.

[31] Fosmark D. S., Torjesen P. A., Kilhovd B. K. (2006). Increased serum levels of the specific advanced glycation end product methylglyoxal-derived hydroimidazolone are associated with retinopathy in patients with type 2 diabetes mellitus. *Metabolism*.

[32] Ishibashi Y., Matsui T., Nakamura N., Sotokawauchi A., Higashimoto Y., Yamagishi S. I. (2017). Methylglyoxal-derived hydroimidazolone-1 evokes inflammatory reactions in endothelial cells via an interaction with receptor for advanced glycation end products. *Diabetes & Vascular Disease Research*.

[33] Lin C. C., Chan C. M., Huang Y. P., Hsu S. H., Huang C. L., Tsai S. J. (2016). Methylglyoxal activates NF-*κ*B nuclear translocation and induces COX-2 expression via a p38-dependent pathway in synovial cells. *Life Sciences*.

[34] Yamawaki H., Saito K., Okada M., Hara Y. (2008). Methylglyoxal mediates vascular inflammation via JNK and p38 in human endothelial cells. *American Journal of Physiology Cell Physiology*.

[35] Schmidt A. M., Yan S. D., Yan S. F., Stern D. M. (2000). The biology of the receptor for advanced glycation end products and its ligands. *Biochimica et Biophysica Acta (BBA) - Molecular Cell Research*.

[36] Martens H. A., Nienhuis H. L. A., Gross S. (2012). Receptor for advanced glycation end products (RAGE) polymorphisms are associated with systemic lupus erythematosus and disease severity in lupus nephritis. *Lupus*.

[37] Hanford L. E., Enghild J. J., Valnickova Z. (2004). Purification and characterization of mouse soluble receptor for advanced glycation end products (sRAGE). *The Journal of Biological Chemistry*.

[38] Geroldi D., Falcone C., Emanuele E. (2006). Soluble receptor for advanced glycation end products: from disease marker to potential therapeutic target. *Current Medicinal Chemistry*.

[39] Raucci A., Cugusi S., Antonelli A. (2008). A soluble form of the receptor for advanced glycation endproducts (RAGE) is produced by proteolytic cleavage of the membrane-bound form by the sheddase a disintegrin and metalloprotease 10 (ADAM10). *The FASEB Journal*.

[40] Pullerits R., Bokarewa M., Dahlberg L., Tarkowski A. (2005). Decreased levels of soluble receptor for advanced glycation end products in patients with rheumatoid arthritis indicating deficient inflammatory control. *Arthritis Research & Therapy*.

[41] Mahajan N., Dhawan V., Malik S., Jain S. (2010). Serum levels of soluble receptor for advanced glycation end products (sRAGE) in Takayasu’s arteritis. *International Journal of Cardiology*.

[42] Ma C. Y., Ma J. L., Jiao Y. L. (2012). The plasma level of soluble receptor for advanced glycation end products is decreased in patients with systemic lupus erythematosus. *Scandinavian Journal of Immunology*.

[43] Park M. J., Lee S. H., Moon S. J. (2016). Overexpression of soluble RAGE in mesenchymal stem cells enhances their immunoregulatory potential for cellular therapy in autoimmune arthritis. *Scientific Reports*.

